# Analgesic prescribing in people with polymyalgia rheumatica: a retrospective cohort study in the Clinical Practice Research Datalink

**DOI:** 10.1093/rap/rkag131

**Published:** 2026-10-23

**Authors:** Helen Twohig, David Jenkinson, James Bailey, Samantha Hider, Ian C Scott, Sara Muller

**Affiliations:** School of Medicine, Keele University, Keele, UK; School of Medicine, Keele University, Keele, UK; School of Medicine, Keele University, Keele, UK; School of Medicine, Keele University, Keele, UK; Haywood Academic Rheumatology Centre, Haywood Hospital, Midlands Partnership University NHS Foundation Trust, Staffordshire, UK; School of Medicine, Keele University, Keele, UK; Haywood Academic Rheumatology Centre, Haywood Hospital, Midlands Partnership University NHS Foundation Trust, Staffordshire, UK; School of Medicine, Keele University, Keele, UK

**Keywords:** polymyalgia rheumatica, prescribing, pain, analgesia, glucocorticoids, primary care

## Abstract

**Objectives:**

To report primary care prescribing rates of analgesics in people with PMR treated with glucocorticoids (GCs), overall and stratified by age, sex and deprivation.

**Methods:**

A sample of >43 000 individuals ≥50 years of age with a coded PMR diagnosis from January 2010 to March 2022 and prescribed GCs within 21 days of the first PMR diagnosis code was constructed in the Clinical Practice Research Datalink Aurum. Prescriptions for ‘all analgesics’, opioids, NSAIDs, gabapentinoids and amitriptyline were defined as prevalent (pre-PMR diagnosis), incident (at diagnosis) or late (post-diagnosis, still GC-treated) and reported overall and stratified by age/sex/deprivation.

**Results:**

While they were receiving GCs for PMR, 56.1% of participants were prescribed an analgesic. Opioids were the most frequently co-prescribed analgesics, followed by NSAIDs. Opioid prescribing was more common in females than males, at older ages and in more deprived areas. Amitriptyline and gabapentinoids were less commonly prescribed but followed the same sex and deprivation trends as opioids. NSAID prescribing was lower with older age, higher for males than females and did not vary with deprivation.

**Conclusion:**

Analgesics are frequently co-prescribed with GCs in people with PMR despite a lack of evidence of effectiveness and risks of adverse events. This shows an unmet need for better pain management in PMR. The identified variation in prescribing of opioids and gabapentinoids by gender/deprivation is consistent with prescribing data for the general population and people with other painful musculoskeletal conditions and suggests that psychosocial and cultural factors affect pain and pain management in patients being treated for PMR. This needs further exploration to address inequalities and reduce iatrogenic harm.

Key messages

- People taking glucocorticoids for PMR are frequently co-prescribed analgesics, particularly opioids, highlighting an unmet need for pain management in this condition.
- Opioids, amitriptyline and gabapentinoids are more commonly prescribed to females, older adults and in areas of greater deprivation but NSAID prescribing does not follow these trends.
- High glucocorticoid and opioid/gabapentinoid co-prescribing rates in PMR are concerning as all these drugs are associated with fractures.

## Introduction

PMR affects older adults, causing significant pain and stiffness and associated functional limitations [1]. The mainstay of treatment is glucocorticoids (GCs), tapered over months to years, which are highly effective in reducing symptoms including pain. However, patients with PMR still experience pain, particularly in the time prior to commencing GCs, during flares before GC dose is adjusted and sometimes with each dose taper [2, 3].

In inflammatory arthritis and other painful long-term musculoskeletal conditions it is known that patients are frequently prescribed opioids, NSAIDS, amitriptyline (used in low doses for pain rather than as an antidepressant) and gabapentinoids, despite there being limited or no evidence of efficacy and substantial evidence of harms [4–6]. Rates of analgesic prescribing are also known to vary by demographic factors and deprivation [7–10], although the complex and varied reasons for this remain underexplored. Analgesics are associated with many adverse outcomes, including the increased risk of falls and fractures with opioids, amitriptyline and gabapentinoids [11, 12] and the increased risk of gastrointestinal adverse events with NSAIDs [13]. People with PMR may be particularly vulnerable to these risks because of older age, disease-related impairment of mobility and confidence and the overlapping adverse effects of GC treatment. The extent of prescribing of these pain medications to people with PMR, and whether there is unwarranted variation in prescribing for people with this condition, is unknown. We aimed to address this using prescribing data from a large national database to explore prescription rates of different classes of analgesia in adults with PMR treated with GCs.

## Methods

We carried out a retrospective cohort study using the Clinical Practice Research Datalink (CPRD) Aurum [14, 15] to assess levels and patterns of co-prescribing of opioids, NSAIDs, amitriptyline and gabapentinoids with GCs in people with PMR in English primary care. The CPRD Aurum contains anonymised, routinely collected data from >1400 English general practices. It is representative of the English population by geographical spread, deprivation, age and sex [16]. The CPRD has Health Research Authority approval to support research using anonymised patient data. This study was approved by the CPRD Research Data Governance Process (ref 23_003646). Individual patient consent is not required, as patients contributing data to the CPRD cannot be identified from the data made available to researchers.

Individuals were included if they had a clinical code for a PMR diagnosis between 1 January 2010 and 31 March 2022, were ≥50 years of age at diagnosis and had prior practice registration for at least 1 year. Individuals were excluded if they had a record of giant cell arteritis diagnosed prior to their PMR or an end-of-life care code at any time. Individuals were required to have had a prescription for prednisolone (the only oral GC specifically licensed in the UK for PMR) within 21 days before or after their first PMR code and a further prednisolone prescription within 90 days of the first. This was based on approaches used to define PMR in previous electronic healthcare records studies [17, 18] but with a shorter time window between prescriptions to reflect updated prescribing practices. However, this definition has not been externally validated. The date of the first prednisolone prescription was considered to be the index date. A continuous prednisolone treatment period was defined as another prescription being issued within 90 days of the previous prescription. Individuals were included in the study if they were treated for a minimum of 90 days and their average daily dose of prednisolone over the first 90 days was at least 3 mg (for details of dose and time-period calculations, see Supplementary Data S1).

Analgesic treatment periods were classified as prevalent (began at least 21 days before the index date and ending no earlier than the index date), incident (began 21 days either side of the index date and ended on or after the index date) or late (began at least 21 days after the index date and before the last prednisolone prescription in the index treatment period) to allow assessment of when in the disease course analgesics were co-prescribed. Proportions of prevalent, incident, late or non-users of amitriptyline, gabapentinoids, NSAIDs, opioids and ‘all analgesics’ (any of these medications) were calculated overall and by age group, Index of Multiple Deprivation (IMD) quintile and sex.

## Results

The cohort contained 43 091 people (see Supplementary Data S1 for eligibility assessment); 43.6% were in the 70–79 years age group and 66.1% were female. A majority were from IMD quintiles 1 and 2 (least deprived), with only 9.6% from quintile 5 (most deprived). Fig. 1 shows the percentage of people in each user category for each medication type. Table 1 details the numbers in each category.

**Figure 1:**
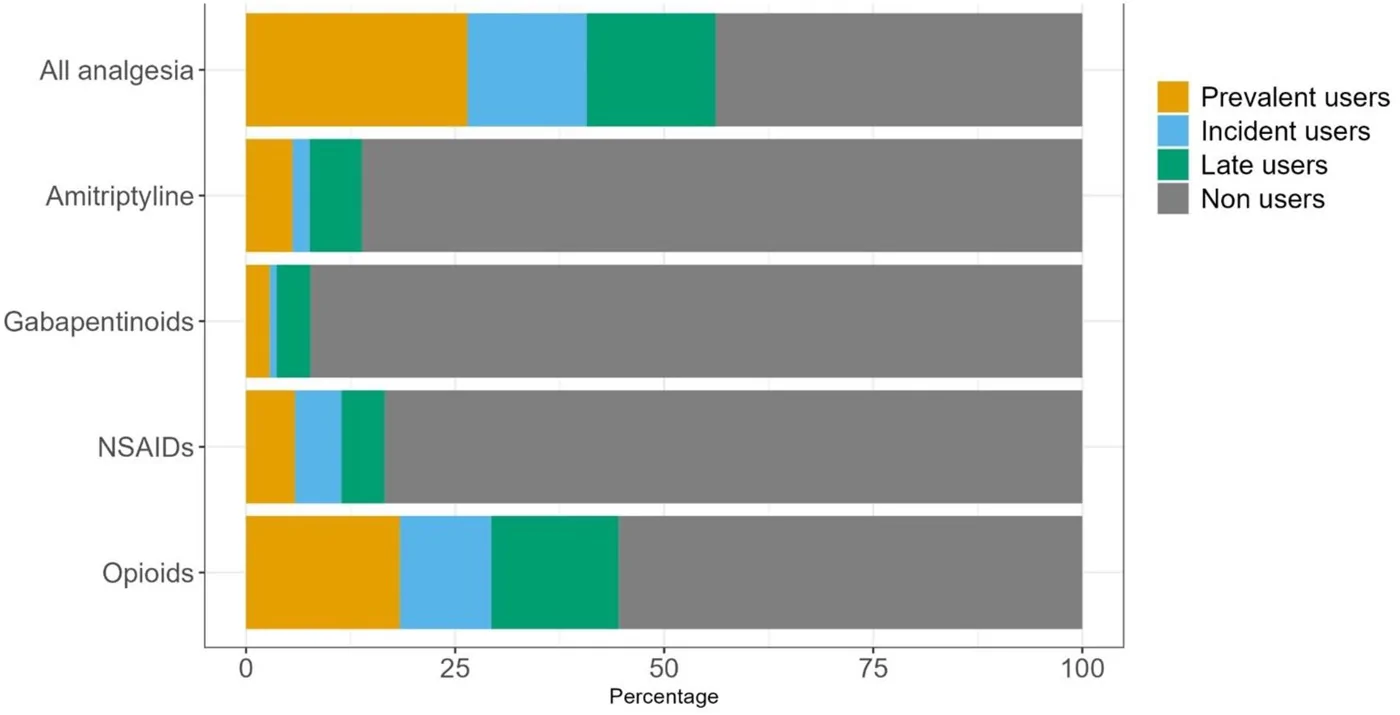
Percentage of prevalent, incident and late users for each medication type Prevalent: began ≥21 days before the index date and ending no earlier than the index date. Incident: began 21 days either side of the index date and ended on or after the index date. Late: began ≥21 days after the index date and before the last prednisolone prescription in the index treatment period

**Table 1:** Analgesia prescription by user category.

Group	Analgesia	*n*	Prevalent			Incident			Late user			Non-user		
			*n*	%	95% CI	*n*	%	95% CI	*n*	%	95% CI	*n*	%	95% CI
Whole cohort	All analgesia	43 091	11 406	26.47	(26.37, 26.57)	6147	14.3	(14.2, 14.4)	6642	15.41	(15.32, 15.51)	18 896	43.85	(43.76, 43.95)
	Amitriptyline	43 091	2394	5.56	(5.50, 5.61)	883	2.0	(2.0, 2.1)	2689	6.24	(6.19, 6.29)	37 125	86.15	(86.10, 86.21)
	Gabapentinoids	43 091	1216	2.82	(2.78, 2.86)	360	0.8	(0.8, 0.9)	1738	4.03	(4.00, 4.07)	39 777	92.31	(92.27, 92.35)
	NSAIDs	43 091	2515	5.84	(5.78, 5.90)	2399	5.6	(5.5, 5.6)	2224	5.16	(5.10, 5.22)	35 953	83.44	(83.38, 83.50)
	Opioids	43 091	7922	18.38	(18.30, 18.48)	4711	10.9	(10.8, 11.0)	6570	15.25	(15.16, 15.34)	23 888	55.44	(55.35, 55.53)
Age 50–59	All analgesia	3590	1014	28.25	(27.94, 28.59)	485	13.5	(13.2, 13.9)	560	15.60	(15.29, 15.94)	1531	42.65	(42.34, 42.99)
	Amitriptyline	3590	250	6.96	(6.80, 7.18)	87	2.4	(2.3, 2.6)	306	8.52	(8.36, 8.74)	2947	82.09	(81.92, 82.30)
	Gabapentinoids	3590	143	3.98	(3.87, 4.12)	28	0.8	(0.7, 0.9)	150	4.18	(4.07, 4.32)	3269	91.06	(90.95, 91.20)
	NSAIDs	3590	362	10.08	(9.86, 10.35)	302	8.4	(8.2, 8.7)	291	8.11	(7.88, 8.37)	2635	73.40	(73.18, 73.67)
	Opioids	3590	616	17.16	(16.88, 17.46)	313	8.7	(8.4, 9.0)	502	13.98	(13.70, 14.28)	2159	60.14	(59.86, 60.44)
Age 60–69	All analgesia	10 424	2807	26.93	(26.74, 27.13)	1602	15.4	(15.2, 15.6)	1534	14.72	(14.52, 14.91)	4481	42.99	(42.80, 43.19)
	Amitriptyline	10 424	601	5.77	(5.66, 5.87)	243	2.3	(2.2, 2.4)	684	6.56	(6.46, 6.67)	8896	85.34	(85.24, 85.45)
	Gabapentinoids	10 424	298	2.86	(2.79, 2.94)	97	0.9	(0.9, 1.0)	399	3.83	(3.76, 3.91)	9630	92.38	(92.32, 92.46)
	NSAIDs	10 424	911	8.74	(8.61, 8.88)	779	7.5	(7.3, 7.6)	683	6.55	(6.42, 6.69)	8051	77.24	(77.10, 77.38)
	Opioids	10 424	1777	17.05	(16.87, 17.23)	1138	10.9	(10.7, 11.1)	1495	14.34	(14.17, 14.53)	6014	57.69	(57.52, 57.88)
Age 70–79	All analgesia	18 786	4867	25.91	(25.76, 26.05)	2682	14.3	(14.1, 14.4)	2821	15.02	(14.87, 15.16)	8416	44.80	(44.66, 44.95)
	Amitriptyline	18 786	978	5.21	(5.13, 5.28)	399	2.1	(2.1, 2.2)	1102	5.87	(5.79, 5.94)	16 307	86.80	(86.73, 86.88)
	Gabapentinoids	18 786	496	2.64	(2.59, 2.70)	155	0.8	(0.8, 0.9)	772	4.11	(4.06, 4.17)	17 363	92.43	(92.37, 92.48)
	NSAIDs	18 786	947	5.04	(4.96, 5.13)	1011	5.4	(5.3, 5.5)	887	4.72	(4.64, 4.81)	15 941	84.86	(84.77, 84.94)
	Opioids	18 786	3447	18.35	(18.22, 18.49)	2088	11.1	(11.0, 11.3)	2833	15.08	(14.95, 15.22)	10 418	55.46	(55.32, 55.60)
Age ≥80	All analgesia	10 291	2718	26.41	(26.22, 26.61)	1378	13.4	(13.2, 13.6)	1727	16.78	(16.59, 16.98)	4468	43.42	(43.22, 43.61)
	Amitriptyline	10 291	565	5.49	(5.39, 5.59)	154	1.5	(1.4, 1.6)	597	5.80	(5.70, 5.90)	8975	87.21	(87.11, 87.31)
	Gabapentinoids	10 291	279	2.71	(2.64, 2.79)	80	0.8	(0.7, 0.9)	417	4.05	(3.98, 4.13)	9515	92.46	(92.39, 92.54)
	NSAIDs	10 291	295	2.87	(2.78, 2.96)	307	3.0	(2.9, 3.1)	363	3.53	(3.44, 3.62)	9326	90.62	(90.54, 90.71)
	Opioids	10 291	2082	20.23	(20.05, 20.42)	1172	11.4	(11.2, 11.6)	1740	16.91	(16.72, 17.10)	5297	51.47	(51.29, 51.66)
IMD quintile 1	All analgesia	12 490	2827	22.63	(22.47, 22.82)	1857	14.9	(14.7, 15.1)	1816	14.54	(14.37, 14.72)	5990	47.96	(47.79, 48.14)
	Amitriptyline	12 490	597	4.78	(4.69, 4.87)	240	1.9	(1.8, 2.0)	756	6.05	(5.96, 6.15)	10 897	87.25	(87.16, 87.34)
	Gabapentinoids	12 490	287	2.30	(2.24, 2.37)	95	0.8	(0.7, 0.8)	409	3.27	(3.22, 3.34)	11 699	93.67	(93.61, 93.73)
	NSAIDs	12 490	695	5.56	(5.46, 5.68)	765	6.1	(6.0, 6.2)	612	4.90	(4.80, 5.01)	10 418	83.41	(83.31, 83.52)
	Opioids	12 490	1811	14.50	(14.35, 14.67)	1345	10.8	(10.6, 10.9)	1769	14.16	(14.01, 14.33)	7565	60.57	(60.42, 60.74)
IMD quintile 2	All analgesia	11 068	2711	24.49	(24.31, 24.69)	1522	13.8	(13.6, 14.0)	1771	16.00	(15.82, 16.20)	5064	45.75	(45.57, 45.95)
	Amitriptyline	11 068	558	5.04	(4.95, 5.15)	231	2.1	(2.0, 2.2)	679	6.13	(6.04, 6.24)	9600	86.74	(86.65, 86.84)
	Gabapentinoids	11 068	271	2.45	(2.39, 2.52)	87	0.8	(0.7, 0.9)	454	4.10	(4.04, 4.18)	10 256	92.66	(92.60, 92.74)
	NSAIDs	11 068	643	5.81	(5.70, 5.93)	594	5.4	(5.3, 5.5)	573	5.18	(5.07, 5.30)	9258	83.65	(83.54, 83.77)
	Opioids	11 068	1813	16.38	(16.21, 16.56)	1159	10.5	(10.3, 10.7)	1738	15.70	(15.53, 15.88)	6358	57.44	(57.27, 57.62)
IMD quintile 3	All analgesia	8786	2314	26.34	(26.13, 26.55)	1213	13.8	(13.6, 14.0)	1387	15.79	(15.58, 16.00)	3872	44.07	(43.87, 44.29)
	Amitriptyline	8786	475	5.41	(5.30, 5.52)	158	1.8	(1.7, 1.9)	563	6.41	(6.31, 6.52)	7590	86.39	(86.28, 86.50)
	Gabapentinoids	8786	234	2.66	(2.60, 2.75)	65	0.7	(0.7, 0.8)	337	3.84	(3.77, 3.92)	8150	92.76	(92.69, 92.85)
	NSAIDs	8786	520	5.92	(5.79, 6.05)	479	5.5	(5.3, 5.6)	455	5.18	(5.05, 5.31)	7332	83.45	(83.33, 83.58)
	Opioids	8786	1631	18.56	(18.37, 18.76)	931	10.6	(10.4, 10.8)	1354	15.41	(15.22, 15.61)	4870	55.43	(55.24, 55.63)
IMD quintile 4	All analgesia	6153	1873	30.44	(30.20, 30.70)	884	14.4	(14.1, 14.6)	979	15.91	(15.67, 16.18)	2417	39.28	(39.04, 39.55)
	Amitriptyline	6153	412	6.70	(6.57, 6.84)	145	2.4	(2.2, 2.5)	377	6.13	(6.00, 6.28)	5219	84.82	(84.69, 84.97)
	Gabapentinoids	6153	218	3.54	(3.45, 3.65)	57	0.9	(0.8, 1.0)	308	5.01	(4.91, 5.12)	5570	90.52	(90.43, 90.63)
	NSAIDs	6153	359	5.83	(5.69, 5.99)	324	5.3	(5.1, 5.4)	328	5.33	(5.18, 5.49)	5142	83.57	(83.42, 83.73)
	Opioids	6153	1367	22.22	(21.99, 22.47)	698	11.3	(11.1, 11.6)	996	16.19	(15.96, 16.44)	3092	50.25	(50.02, 50.51)
IMD quintile 5	All analgesia	4131	1552	37.57	(37.28, 37.89)	601	14.5	(14.3, 14.9)	615	14.89	(14.60, 15.21)	1363	32.99	(32.70, 33.32)
	Amitriptyline	4131	326	7.89	(7.72, 8.08)	105	2.5	(2.4, 2.7)	278	6.73	(6.56, 6.91)	3422	82.84	(82.67, 83.02)
	Gabapentinoids	4131	188	4.55	(4.43, 4.71)	55	1.3	(1.2, 1.5)	214	5.18	(5.06, 5.34)	3674	88.94	(88.82, 89.09)
	NSAIDs	4131	271	6.56	(6.39, 6.76)	213	5.2	(5.0, 5.4)	229	5.54	(5.37, 5.75)	3418	82.74	(82.57, 82.94)
	Opioids	4131	1209	29.27	(28.98, 29.58)	520	12.6	(12.3, 12.9)	648	15.69	(15.40, 16.00)	1754	42.46	(42.17, 42.77)
Male	All analgesia	16 565	3895	23.51	(23.36, 23.67)	2635	15.9	(15.8, 16.1)	2191	13.23	(13.08, 13.38)	7844	47.35	(47.20, 47.51)
	Amitriptyline	16 565	567	3.42	(3.36, 3.50)	290	1.8	(1.7, 1.8)	695	4.20	(4.13, 4.27)	15 013	90.63	(90.56, 90.70)
	Gabapentinoids	16 565	361	2.18	(2.13, 2.24)	119	0.7	(0.7, 0.8)	505	3.05	(3.00, 3.11)	15 580	94.05	(94.01, 94.11)
	NSAIDs	16 565	1083	6.54	(6.44, 6.64)	1071	6.5	(6.4, 6.6)	776	4.68	(4.59, 4.78)	13 635	82.31	(82.22, 82.41)
	Opioids	16 565	2672	16.13	(15.99, 16.27)	2011	12.1	(12.0, 12.3)	2115	12.77	(12.63, 12.91)	9767	58.96	(58.82, 59.11)
Female	All analgesia	26 526	7511	28.32	(28.19, 28.44)	3512	13.2	(13.1, 13.4)	4451	16.78	(16.66, 16.91)	11 052	41.66	(41.54, 41.79)
	Amitriptyline	26 526	1827	6.89	(6.82, 6.96)	593	2.2	(2.2, 2.3)	1994	7.52	(7.45, 7.59)	22 112	83.36	(83.29, 83.43)
	Gabapentinoids	26 526	855	3.22	(3.17, 3.27)	241	0.9	(0.9, 1.0)	1233	4.65	(4.60, 4.70)	24 197	91.22	(91.17, 91.27)
	NSAIDs	26 526	1432	5.40	(5.33, 5.47)	1328	5.0	(4.9, 5.1)	1448	5.46	(5.39, 5.53)	22 318	84.14	(84.06, 84.21)
	Opioids	26 526	5250	19.79	(19.68, 19.91)	2700	10.2	(10.1, 10.3)	4455	16.79	(16.68, 16.91)	14 121	53.23	(53.12, 53.35)

IMD data were missing in 465 participants.

A total of 56.1% were prescribed at least one type of analgesic while they were also prescribed prednisolone, 26.5% of the sample were prevalent users of any analgesia, 14.3% were incident users and 15.4% were late users.

The most prescribed analgesics were opioids, prescribed to 44.6% while they were also prescribed prednisolone (18.4% prevalent, 10.9% incident and 15.2% late). For NSAIDs, proportions of prevalent, incident and late use were similar at ≈5%. For amitriptyline, proportions of prevalent and late use were similar (5.6% and 6.2%, respectively) but incident prescribing was less (2.0%). Gabapentinoids were the least prescribed group and, where they were used, the proportion of late users (4.0%) was higher than that of prevalent (2.8%) or incident users (0.8%).

When stratified by age, all analgesic prescribing showed little variation. However, opioid prescribing was higher at older ages (39.9% in ages 50–59 years *vs* 48.5% in ages ≥80 years) and NSAID prescribing was less (26.6% in ages 49–59 years *vs* 9.4% in ages ≥80). Amitriptyline prescribing was also lower at older ages but the trend was less marked than for NSAIDs. Females were prescribed each category of analgesic more commonly than males (58.3% *vs* 52.6% for all analgesics, 46.8% *vs* 41.0% for opioids, 16.6% *vs* 9.4% for amitriptyline and 8.8% *vs* 5.9% for gabapentinoids) except for NSAIDs, which were prescribed to 17.7% of males compared with 15.9% of females. Prescribing of all analgesics (52.0% in IMD 1 *vs* 67.0% in IMD 5), opioids (39.4% in IMD 1 *vs* 57.5% in IMD 5), amitriptyline (12.8% in IMD 1 *vs* 17.2% in IMD 5) and gabapentinoids (6.3% in IMD 1 *vs* 11.1% in IMD 5) was more frequent with higher levels of deprivation. There was little variation in NSAID prescribing across IMD quintiles.

## Discussion

This is the first study to look at prescribing of analgesics in people with PMR currently treated with GCs and included a cohort of >43 000 people from a large national database. We show that more than half of people with PMR are co-prescribed analgesics and prednisolone. Opioids are most frequently prescribed and prescribing of these is more common in females, at older ages and with higher deprivation. NSAIDs are prescribed less often, but in contrast to all other categories of analgesia are prescribed to a higher proportion of males than females. NSAID prescriptions were less frequent at older ages and do not appear to vary with deprivation. Amitriptyline and gabapentinoids are not commonly co-prescribed with GCs, but when considered by gender and deprivation, show similar trends to those of opioids.

One previous study looked at opioid prescribing in an established PMR cohort compared with a matched general population group in the USA [19]. They found no difference in either incident or chronic opioid use. However, this was long-term follow up data from people diagnosed with PMR between 1970 and 2014, reporting opioid prescribing annually from 2005 to 2015. Our findings, in contrast, relate to people with active PMR on current treatment with GCs and provide the first picture of this prescribing in England.

The rates of opioid prescribing identified in this cohort are similar to those reported in a recent national English study of analgesia prescribing in inflammatory arthritis [4]. NSAID prescribing, however, is significantly lower, which may be explained by the older age of the PMR population given the well-established risks of NSAIDs in this age group. Public Health England data [20] from 2017–18 shows that 13% of the general adult population were prescribed opioids and 3% were prescribed gabapentinoids. Gabapentinoid use in our study population is therefore similar to general population levels. However, opioid use is slightly higher than in the general population prior to PMR diagnosis and increases further around the time of diagnosis and afterwards.

It is not possible to discern from these observational data whether the prescribed analgesic is for PMR symptoms or for other conditions. However, by examining prescribing in relation to time of diagnosis, some interpretation of this can be inferred. Incident prescriptions (first initiated within 21 days either side of the first GC prescription) suggest that analgesia was required at the time that PMR was being considered/diagnosed. Late prescriptions indicate pain later in the disease course, either from PMR or from another condition, that was not suppressed by the prednisolone treatment. This suggests there may be an unmet need regarding pain management in people with PMR and is of concern as the high co-prescription rates of GCs and analgesia (particularly opioids) raise the risk of additive adverse effects such as falls and fractures.

The variation identified in prescribing by sex and deprivation is also in line with findings from studies of analgesic prescribing in the general population. Many previous studies [7–9, 20] have reported an association between higher opioid and gabapentinoid prescribing levels and higher levels of deprivation. Previous studies have also shown that women are prescribed more analgesia than men [21, 22]. Higher rates of analgesic prescribing in females and in people living in areas of higher deprivation may be explained in part by a higher prevalence of painful conditions and a higher prevalence of multiple pain conditions occurring simultaneously in females and higher rates of chronic pain in people living in deprivation [23]. However, many other variables linked to sex and deprivation may also influence analgesic prescribing, including psychosocial factors, healthcare use behaviours, cultural expectations, prevalence of mental health conditions and clinician bias. Our findings—that in PMR, prescribing of opioids, gabapentinoids and amitriptyline is higher in females than males and in those living in areas of higher deprivation—are therefore consistent with previous research. However, importantly, we show that this variation exists within prescribing for patients with a single condition. It is unclear whether this variation is an appropriate response to differences in severity of pain from PMR or comorbid conditions or represents unwarranted variations in pain management. However, given the limited evidence of effectiveness of these medications for chronic pain, it may still represent inequality in the level of exposure to medication-related harm.

NSAID prescribing shows an interesting exception to these prescribing patterns, with higher prescription rates in men than women and no variation with deprivation. The reasons for this are unclear and require qualitative exploration.

Strengths of this work include the large, representative dataset and the consideration of multiple classes of analgesics. However, there are limitations related to the use of observational data. First, PMR is a difficult diagnosis to make with no single diagnostic test and there is therefore the potential for misclassification. The approach we took was based on that used in previous studies of PMR that used electronic healthcare records data [17, 18] with a requirement for a prescription for prednisolone within 21 days of the first PMR code and a further prescription within 90 days of that, but it is still possible that there was some misclassification in our study population. Second, while the analgesic prescriptions were during the time that participants were on GCs for PMR, the indication for the analgesia is not known. This makes it difficult to assess the appropriateness of prescribing but does not detract from the importance of the finding of high rates of prescribing of these medications among people co-prescribed prednisolone.

In conclusion, this study, which is the first to look at analgesic prescribing in English primary care for people co-prescribed prednisolone for PMR, has found high rates of opioid prescribing and variations in analgesic prescribing by age, sex and deprivation. This is despite the known risks of these medications, which may add to and interact with risks of prednisolone treatment in this older population. Pain management in people with PMR requires further attention and exploration of the reasons for the observed prescribing patterns to address these issues.

## Supplementary Material

rkag131_Supplementary_Data

## Data Availability

Data are not publicly available, as they were obtained from the CPRD. CPRD data governance does not allow us to distribute patient data to other parties. Researchers may apply for data access at http://www.CPRD.com/.
