# Supplementary material for: Analgesic prescribing in people with polymyalgia rheumatica: a retrospective cohort study in the Clinical Practice Research Datalink
Source: Rheumatol Adv Pract. 2026 Oct 23;10(4):rkag131. doi: 10.1093/rap/rkag131 (PMC13750171; doi:10.1093/rap/rkag131)
Supplement: Supplementary file 1 [file rkag131_supplementary_data.docx]

**Supplementary Data S1 for:** Analgesic prescribing in people with polymyalgia rheumatica: a retrospective cohort study in the Clinical Practice Research Datalink

# **Calculating average daily dose of prednisolone**

The amount of prednisolone in each prescription was calculated by multiplying the substance strength (in mg) by the quantity of tablets. Where multiple prescriptions (different strengths) were issued on the same date, these were summed. For each person the amount of prednisolone in each prescription that was issued within 90 days of their index date was added together. The time covered by these combined prescriptions was calculated as the time from index date to the date of the person’s first prescription that was more than 90 days after index date. The total amount of prednisolone was divided by this time to give the average daily dose in mg.

# **Calculating continuous treatment periods for other medications**

Prescription duration was taken from the duration field in the CPRD Aurum Drug Issue file. If duration was negative, zero or greater than 90 days it was imputed as the mean duration for that type of medication. Each prescription’s end date was calculated as its index date plus its duration. A calculated break between the end of one prescription and the start of another of >90 days was considered the end of that treatment period and any further prescription after this date a new treatment period. The treatment period’s end date was the latest prescription end date within that treatment period, or the earliest of a person’s death date, registration end date and last collection date, whichever the earlier.

Prescriptions for two or more medications of the same type (on the same codelist) were considered to be a continuation of the prescription.

**Table 1: Eligibility assessment process**

| Number of people in table received from CPRD |  | 66,749 |
| --- | --- | --- |
| Number of people with Northern Ireland practice code | 181 |  |
| Number of people with death date, registration end date or last collection date before PMR index date | 5 |  |
| Number of people with an observation with a missing observation date | 209 |  |
| Number of people with an end of life or palliative care observation | 4,393 |  |
| Number of people with a GCA observation before their PMR observation | 1,504 |  |
| Number of people without a prednisolone prescription | 1,306 |  |
| Number of people without a prednisolone prescription within 21 days of PMR consultation | 4,672 |  |
| Number of people without a further prednisolone prescription within 90 days of first prescription | 2,769 |  |
| Number of people whose index prednisolone treatment period does not last more than 90 days | 8,262 |  |
| Number of people whose average daily dose over first 90 days was not more than 3mg | 357 |  |
| Number of people included in the study |  | 43,091 |
